# Quality of care provided to patients with type 2 diabetes mellitus in Tshwane, South Africa

**DOI:** 10.4102/phcfm.v16i1.4576

**Published:** 2024-07-31

**Authors:** Ntlogeleng M. Mogale, Thembelihle S. Ntuli, Thembekile S. Dhlamini, Paul K. Chelule

**Affiliations:** 1 Department of Public Health, Faculty of Health Care Sciences, Sefako Makgatho Health Sciences University, Pretoria, South Africa; 2 Department of Statistical Sciences and Operational Research, Faculty of Science and Technology, Sefako Makgatho Health Sciences University, Pretoria, South Africa

**Keywords:** type 2 diabetes, quality of care, treatment guidelines, screening, equipment, patient education

## Abstract

**Background:**

Type 2 diabetes mellitus (T2D) is a public health challenge, affecting 90% of all patients with diabetes, globally. Compliance to treatment guidelines among healthcare professionals (HCPs) is low, thus resulting in inadequate quality of patient care and poor health outcomes among patients.

**Aim:**

To examine the availability of equipment, guidelines, screening and education offered to patients with T2D and compare between clinics and community health centres (CHCs).

**Setting:**

Tshwane Metropolitan Municipality, Gauteng Province, South Africa.

**Methods:**

A cross-sectional descriptive study utilised a self-administered questionnaire to collect data from nurses and doctors responsible for treating patients with T2D, from May to June 2022. About 250 eligible HCPs were recruited during routine morning meetings in 22 clinics and six CHCs.

**Results:**

More than 80% of HCPs reported having basic equipment except for ophthalmoscopes, Snellen charts (67%), tuning forks (64%), electrocardiograms (ECG) (46%) and monofilaments (12%). SEMDSA guidelines were reported by 16% of the participants, Diabetic Foot Care Guidelines were reported by 54% and Dietary Guidelines for Diabetic Patients by 55%. Furthermore, 91%, 71% and 69% of HCPs reported that ECG, microalbumin-creatinine and foot examinations were not always performed, respectively. About 66% and 17% always offered individual educational and group sessions, respectively.

**Conclusion:**

Equipment availability and compliance with treatment guidelines, patient education and screening of chronic complications are inadequate.

**Contribution:**

The study highlights the poor adherence to treatment guidelines and inadequate equipment in health facilities. These shortcomings could lead to missed opportunities for early diagnosis of complications and ultimately poorer patient outcomes.

## Introduction

Type 2 diabetes mellitus (T2D) is a public health crisis accounting for approximately 90% of all patients with diabetes, globally.^1^ As of 2021, the global prevalence of T2D was estimated at 10.5%, and projected to rise to 12.2% (783 million individuals) by 2045.^1^ This concerning trend is particularly pronounced in developing nations where rapid economic growth and urbanisation are fuelling the disease burden, and disproportionately impacting urban populations compared to their rural counterparts.^2,3,4^ Numerous factors contribute to the increased risk of T2D, including unhealthy dietary patterns characterised by high amounts of sugar intake, consumption of processed foods, overweight and obesity, certain ethnicities, family history and sedentary lifestyles.^1,5,6^

In South Africa, T2D remains a significant challenge,^7,8,9^ despite established treatment guidelines to achieve optimal care for patients.^10,11,12^ Hospital-based studies have shown that compliance to these guidelines is a major challenge.^13,14,15^ This issue permeates into primary healthcare (PHC) clinics that also struggle to deliver adequate care and screening for complications in patients living with T2D.^16,17^ Inadequate resources, including the lack of readily available guidelines, educational materials and some screening equipment, exacerbate the situation.^17^

Complications related to diabetes are associated with poor glycaemic control, and therefore early glucose management and control is crucial in decreasing the occurrence and progression of complications such as cardiovascular, microvascular and macrovascular complications and ultimately death.^1,11,18,19,20^ Several studies have shown that glycaemic control in patients living with T2D is also suboptimal, with the proportion of patients with controlled glycaemia not exceeding 30% in most settings.^16,20,21,22,23^ Notwithstanding the importance of glycaemic control, treatment should also focus on other concomitant risk factors as well as regular screening of both acute and chronic complications.^1,11,20^ Noncompliance with treatment guidelines, poor glycaemic control from patients themselves, healthcare professionals (HCPs) and the healthcare system, can have devastating health outcomes.

In South Africa, clinics are the first point of entry in the healthcare system, and they are led mainly by nurses, with few medical practitioners and some of whom perform sessional work while community health centres (CHCs) have full-time doctors.^16,17^ It is important to understand the quality of care they provide to patients living with T2D and the challenges they face when performing their duties. There is a paucity of data regarding the availability of resources and management of patients living with T2D. Therefore, the aim of this study was to examine the availability of resources and care provided to patients living with T2D by HCPs and make a comparison between clinics and CHCs, in Tshwane.

## Materials and methods

### Study design

This was a cross-sectional descriptive study using a standardised self-administered questionnaire to collect data from HCPs. Data were collected over an 8-week period, from May to June 2022.

### Study setting

The study was conducted in Tshwane Metropolitan Municipality, one of the three metros in Gauteng Province of South Africa. There are 107 clinics and 8 CHCs in the district. This study was conducted in 22 clinics and 6 CHCs. These PHC facilities offer free health services to the users.

### Study population, sample size and sampling strategy

The study population consisted of nurses and medical doctors responsible for the management and treatment of patients living with T2D and who had at least 1 year of clinical experience in the management of patients. The Cochran’s formula was used to calculate the minimum sample size required. The following parameters were used: 5% margin of error, 95% confidence interval, 80% response rate and an estimated number of about 2052 HCPs (personal communication). The minimum sample size calculated for this study was 246. To select the facilities, five of the seven regions in the district were randomly selected to form clusters. In each cluster, a proportionate number of facilities was randomly selected to ensure representativeness. Consecutive sampling technique was used to select the participants who were available in facilities during the period of data collection.

### Data collection tool, procedure and description of variables

A 35-item self-administered questionnaire, developed from existing literature and validated by a panel of five experts, was used to collect data.^17,18^ The questionnaire consisted of four sections, namely sociodemographic profile, availability of equipment and treatment guidelines, types and frequency of screening tests performed, and education provided to patients living with T2D. Following a pilot test with no modifications, the questionnaire was administered to 250 eligible HCPs recruited mainly in groups, during their morning routine meetings at their respective facilities. The study purpose, participant rights and confidentiality were explained prior to participation. Verbal consent was obtained before participants completed the questionnaire, which took less than 20 min.

Description of the sociodemographic variables can be obtained from this substudy in Tshwane District.^24^ In this study, staff categories included nurses (professional nurses, staff nurses, assistant nurses and student nurses) and doctors (family physicians, medical officers, registrars, community service doctors and interns). Participants were asked if they had medical equipment and treatment guidelines in their respective facilities and these were measured as either ‘Yes’ or ‘No’ and coded as 1 or 0, respectively. A three-point Likert scale was used to assess the frequency of tests performed and training provided to patients, and these were categorised as ‘Never’, ‘Sometimes’ and ‘Always’, coded as 0, 1 and 2, respectively.

### Data analysis

Google Forms (Google, LLC) was used to capture data which were automatically populated onto a linked Google Sheets (Google, LLC). After capturing, data were downloaded and imported into STATA 17 SE (College Station, TX: StataCorp LLC) for cleaning, coding and analysis. Descriptive statistics was used to analyse data and the results are presented using frequency tables and charts. Comparison between clinics and CHCs was performed using chi-squared test, and a *p*-value of less than 0.05 was considered statistically significant.

### Ethical considerations

An application for full ethical approval was made to the Sefako Makgatho Health Sciences University Research Ethics Committee and ethics consent was received on 11 November 2021. The ethics approval number is SMUREC/H/318/2021:PG. The study was conducted in accordance with the Declaration of Helsinki. The study information and rights to voluntary participation and withdrawal were presented to participants. Privacy, anonymity and confidentiality were assured. The questionnaire was anonymous and no identifiable information of the participants was collected. Participants preferred consenting verbally prior to completing the questionnaires.

## Results

### Participants’ characteristics

Of the 250 questionnaires distributed, only 205 were received from the participants, thus constituting a response rate of 82%. The distribution of participants per facility type is shown in Table 1. Most participants were nurses (84%, *n* = 173), and of those, 91% (*n* = 158) were professional nurses. Medical doctors accounted for 16% (*n* = 32) of the participants, and of those, 47% (*n* = 15) were medical officers (MO) or registrars, and 38% (*n* = 12) were community service doctors or interns. Eighty percent (*n* = 165) of the participants were stationed in clinics. There were significantly more nurses in clinics than CHCs (84%, *n* = 146 vs. 16%, *n* = 27; *p* < 0.001), whereas the distribution of medical practitioners was not statistically significant between the two types of health facilities (59%, *n* = 19 vs. 41%, *n* = 13; *p* = 0.1533). More details on the sociodemographic characteristics can be obtained from this substudy in Tshwane District.^24^

**TABLE 1:** Distribution of healthcare professionals between primary healthcare facilities and community health centres in Tshwane.

Healthcare professionals	*n*	%	Facility type			
			Clinics (*N* = 165)		CHCs (*N* = 40)	
			*n*	%	*n*	%
**Nurses (all)**	173	-	146	-	27	-
Professional nurses	158	91	134	85	24	15
Staff nurses, assistant nurses and student nurses	15	9	12	80	3	20
**Medical doctors (all)**	32	-	19	-	13	-
Family physician	5	15	2	40	3	60
Medical officers and registrar	15	47	7	47	8	53
Community service and intern doctors	12	38	10	83	2	17

CHC, community health centre.

### Availability of medical equipment and guidelines

More than 80% of HCPs reported to have basic medical equipment in their respective facilities. Ophthalmoscopes were reported to be available by 71% of HCPs, Snellen charts by 67%, tuning forks by 64%, electrocardiogram (ECG) machines by 46% and monofilaments by 12%, as presented in Table 2. The results further show that most clinics were better equipped compared to CHCs (*p* < 0.05). There were, however, a comparable shortage of ophthalmoscopes (*p* = 0.375) and Snellen charts (*p* = 0.307) between the two categories of health facilities (Table 2).

**TABLE 2:** Availability of basic essential equipment in clinics and community health centres.

Type(s)	Availability of equipment				Facility type								*p*-value
					Clinics (*N* = 165)				CHCs (*N* = 40)				
	Yes	%	No	%	Yes	%	No	%	Yes	%	No	%	
Scale and height rod	186	91.0	19	9.0	153	93.0	12	7.0	33	82.5	7	18.5	0.045
Tape measure	185	90.0	20	10.0	153	93.0	12	7.0	32	80.0	8	20.0	0.015
Glucometers	198	97.0	7	3.0	162	98.0	3	2.0	36	90.0	4	10.0	0.011
Blood pressure machine	193	94.0	12	6.0	161	98.0	4	2.0	32	80.0	8	20.0	< 0.001
Obese cuffs	174	85.0	31	15.0	145	88.0	20	12.0	29	73.0	11	27.0	0.015
ECG machine	94	46.0	111	54.0	83	50.3	82	49.7	11	28.0	29	72.0	0.009
Ophthalmoscope	145	71.0	60	29.0	119	72.0	46	28.0	26	65.0	14	35.0	0.375
Snellen charts	137	67.0	68	33.0	113	68.0	53	32.0	24	60.0	16	40.0	0.307
Tuning forks	132	64.0	73	36.0	115	70.0	50	30.0	17	43.0	23	57.0	0.001
Patella hammer	176	86.0	29	14.0	147	89.0	18	11.0	29	73.0	11	27.0	0.007
Monofilament	25	12.0	180	88.0	24	15.0	141	85.0	1	3.0	39	97.0	0.037

ECG, electrocardiograms; CHC, community health centres.

Table 3 depicts the availability of guidelines in the PHC facilities as reported by HCPs. Overall, less than two-thirds of the participants indicated to have the Society for Endocrinology, Metabolism and Diabetes of South Africa (SEMDSA) Guidelines (16%), Foot Care Guidelines (54%) and Dietary Guidelines for Diabetic Patients (55%). The availability of the Essential Drug List (EDL) and the Department of Health Guidelines for Primary Care of Diabetics guidelines was reported by 83% and 73% of HCPs, respectively. The aforementioned guidelines as well as the Foot Care Guidelines were significantly found in clinics than CHCs (*p* < 0.05). The availability of the SEMDSA Guidelines and Dietary Guidelines for Diabetic Patients was similar between clinics and CHCs (*p* > 0.05).

**TABLE 3:** Availability of guidelines.

Treatment guidelines	Available		Clinics				CHCs				*p*-value
	*n*	%	Yes	%	No	%	Yes	%	No	%	
SEMDSA	32	16.0	27	16.0	138	84.0	5	13.0	35	87.0	0.546
Essential Drug List	171	83.0	142	86.0	23	14.0	29	73.0	11	27.0	0.039
Department of Health Guidelines for Primary Care for Diabetes	149	73.0	125	76.0	40	24.0	24	60.0	16	40.0	0.045
Foot Care Guideline	111	54.0	95	58.0	70	42.0	16	40.0	24	60.0	0.045
Dietary Guidelines for Diabetic Patients	113	55.0	90	55.0	75	45.0	23	58.0	17	42.0	0.736

SEMDSA, Society for Endocrinology, Metabolism and Diabetes of South Africa; CHC, community health centre.

### Tests performed for patients living with type 2 diabetes mellitus

The participants were asked how often they performed certain tests as part of the management, care and monitoring of patients living with T2D (Table 4). Overall, around one-third of the participants reported that they always performed foot examination (31%) during every visit, along with eye examinations (36%) and nerves or pedal pulse (34%). Regarding HBA1c testing, 61% of the participants reported to always perform the test every 6 months. Microalbumin-creatinine ratio and ECG tests were reported to be consistently performed by 29% and 9% of the HCPs, respectively. Clinics were significantly more likely to always perform foot examinations at every visit than CHCs (35% vs. 15%, *p* < 0.05). Apart from foot examination, no statistically significant difference was observed in the performance of the tests between clinics and CHCs (*p* > 0.05). Although slightly more HCPs in CHCs reported to perform eye examinations and microalbumin-creatinine ratio test than in the clinics, the difference was not statistically significant. The tests were categorised into acute and chronic complications. Results showed that screening for acute complications is always performed as per guidelines relative to chronic complications (Table 4).

**TABLE 4:** Frequency of tests performed for the management and care for patients living with type 2 diabetes mellitus between primary healthcare facilities and community health centres.

Tests performed	All						Clinics						CHCs						*p*-value
	Never		Sometimes		Always		Never		Sometimes		Always		Never		Sometimes		Always		
	*n*	%	*n*	%	*n*	%	*n*	%	*n*	%	*n*	%	*n*	%	*n*	%	*n*	%	
Signs and symptoms¶, *	12	6.0	29	14.0	164	80.0	8	5.0	23	14.0	134	81.0	4	10.0	6	15.0	30	75.0	0.440
Weigh patient¶, *	10	5.0	17	8.0	178	87.0	6	4.0	13	8.0	146	88.0	4	10.0	4	10.0	32	80.0	0.211
Blood pressure¶, *	9	4.0	4	2.0	192	94.0	6	4.0	3	2.0	156	94.0	3	8.0	1	2.0	36	90.0	0.537
Foot examination¶, †	29	14.0	112	55.0	64	31.0	19	12.0	88	53.0	58	35.0	10	25.0	24	60.0	6	15.0	0.014
Urine glucose/ketones¶, *	11	5.0	66	32.0	128	62.0	8	5.0	52	31.0	105	64.0	3	7.0	14	35.0	23	58.0	0.692
HBA1c‡	21	10.0	58	28.0	126	61.0	15	9.0	49	30.0	101	61.0	6	15.0	9	23.0	25	62.0	0.428
Proteinuria/MACR§, †	34	17.0	49	24.0	122	59.0	25	15.0	40	24.0	100	60.0	9	22.5	9	22.5	22	55.0	0.533
Urea and creatinine§, †	12	6.0	24	12.0	169	82.0	7	4.0	18	11.0	140	85.0	5	12.0	6	15.0	29	73.0	0.090
Eye examination§, †	40	20.0	90	44.0	75	36.0	29	18.0	78	47.0	58	35.0	11	28.0	12	30.0	17	42.0	0.117
Nerves/pedal pulse§, †	59	29.0	77	37.0	69	34.0	47	29.0	61	37.0	57	34.0	12	30.0	16	40.0	12	30.0	0.860
Lipid testing§, †	29	15.0	28	13.0	148	72.0	21	13.0	20	12.0	124	75.0	8	20.0	8	20.0	24	60.0	0.158
Microalbumin-creatinine ratio (MACR)§, †	99	48.0	47	23.0	59	29.0	84	51.0	36	22.0	45	27.0	15	38.0	11	27.0	14	35.0	0.313
ECG§, †	147	71.0	40	20.0	18	9.0	121	73.0	29	18.0	15	9.0	26	65.0	11	27.0	3	8.0	0.362

ECG, electrocardiograms; CHC, community health centre.

* , test for acute complications;

† , test for chronic complications;

‡ , Performed every 6 months;

§ , Performed annually;

¶ , Performed at every visit.

### Education of patients living with type 2 diabetes mellitus

**FIGURE 1:**
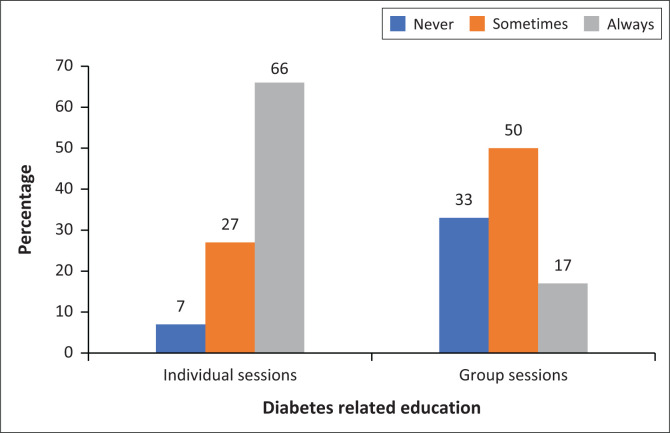
Proportion of healthcare professionals who reported to have individual sessions and group sessions for patients living with type 2 diabetes mellitus in their facilities.

With regard to the frequency of education provided to patients living with T2D, most HCPs (66%) indicated to always offer individual educational sessions compared to 27% of those who reported to sometimes provide such sessions (Figure 1). No statistically significant difference was observed between the clinics and CHCs in this regard (*p* > 0.05).

In terms of group sessions held with patients (Figure 1), 50% of HCPs reported that they sometimes held group sessions with patients and only 17% reported to always have such sessions. Again, no significant difference was observed between the clinics and CHCs (*p* > 0.05). Even though the results are not shown, 76% of the participants indicated that nurses were mainly responsible for health education of patients with diabetes and at times, medical doctors (14%), health promoters (8%) and diabetes educators (2%) would offer such education to patients.

## Discussion

This study aimed to determine the care and management of patients living with T2D focusing on the availability of equipment, diabetes management guidelines, frequency of screening tests performed and provision of educational sessions to patients.

### Availability of equipment

In this study, most participants reported to have scale and height rods, tape measures, glucometers, blood pressure machines and obese cuffs. Fewer participants, however, reported to have monofilaments, ECG machines, tuning forks, Snellen charts and ophthalmoscopes. In the Tshwane District, several studies assessed PHC clinics’ capacity and quality of care provided to patients with diabetes, as outlined in the National Diabetes Guidelines of South Africa.^11,12,13^ A study by Webb and colleagues in Tshwane assessed the ability of PHC clinics to provide quality diabetes care and found that most essential equipment for the care of patients with diabetes were available in the participating clinics, and that 58% had ECG machines, 75% had ophthalmoscopes, and 66.7% had Snellen charts and tuning forks.^17^ No meaningful change is observed in terms of the availability of medical equipment between the previous studies and the current study. In the present study, 12% of the participants reported to have monofilaments in their facilities. The reason for the lack of monofilaments in these studies could be that this medical equipment is unknown among many PHC professionals, and therefore training of HCPs should be considered.^17,25^

A multicentre study conducted in three districts in South Africa found that primary eye care has many challenges which include organisation of care, resource availability and clinical competence.^26^ Similarly, in a Malawian study, ophthalmoscopes and Snellen charts were found in 20% of the clinics and none had monofilaments.^27^ Another study in Kinshasa also showed that monofilaments were available in 20% of the clinics.^25^

When comparing clinics and CHCs regarding the availability of equipment, our results suggest that the clinics seem to have the most medical equipment. This finding was unexpected as it would have been more reasonable if the opposite were true because of the size, number of staff and patients seen at CHCs compared to clinics.^28^ Moreover, when the findings of this study are compared with those conducted in the same health district, this raises concerns of the status quo that has not changed over the years, especially with regard to equipment and screening for chronic complications.^17^

### Availability of guidelines

In the current study, only 16% of the participants reported to have the SEMDSA Guidelines in their facilities, while Diabetic Foot Care Guidelines and Dietary Guidelines for Diabetic Patients were reported to be available by 54% and 55% of the study participants, respectively. There are various guidelines for the management of patients living with T2D in South Africa; however, studies have shown that most of these guidelines are not available in some of the PHCs.^10,11,12^ A multicentre study conducted in three South African provinces, that is, KwaZulu-Natal, Eastern Cape and Free State, found no facility having the updated Management of T2D in Adults at Primary Health Care Guidelines.^29^ Another study in Tshwane found that none of the study participants knew about the SEMDSA Guidelines, and furthermore, Diabetic Foot Care Guidelines and Dietary Guidelines for Diabetes Patients were found in 25% and 8% of the clinics, respectively.^17^

In this study, the EDL and the Department of Health Guidelines for Primary Care for Diabetes were reported to be available by 83% and 73% of HCPs, respectively. A study in Tshwane found that all facilities had EDL; however, only 58% had the Department of Health Guidelines for Primary Care for Diabetes.^17^ In a study conducted at Ga-Dikgale Health and Demographic Surveillance Site in Limpopo Province in South Africa, it was found that poor dissemination of guidelines in clinics contributed to a lack of knowledge among nurses.^30^ Most patients living with T2D are managed at PHC facilities, and if guidelines are disseminated and properly used, they can assist HCPs to better control many noncommunicable diseases.^29^

### Screening tests performed on patients living with type 2 diabetes mellitus

In this current study, HCPs reported that ECG, microalbumin-creatinine, foot examinations, nerve pulse, eye examinations and lipid testing are not always done in their facilities, despite the availability of guidelines. Several hospital-based studies in South Africa have shown that screening of patients living with T2D for complications in accordance with the guidelines was not always done. A study conducted at a regional hospital in KwaZulu-Natal Province, where 750 files of patients with T2D were reviewed, found that only 6% of the patients had their feet examined, 3.9% were tested for microalbuminuria and 43% had at least one eye examination.^13^ Another study which reviewed 500 files of patients with T2D at a district hospital in the same province reported that 7.8%, 13.6%, 10.4% and 13.2% of the patients had foot examination, eye examination, microalbumin-creatinine ratio test and ECG tests performed, respectively.^14^ Out of 1340 patient files reviewed at a provincial hospital in Northwest Province, eye examination was performed in only 19.5% of the patients, foot examination in 20.6% and microalbumin-creatinine ratio test in only 1.1%.^15^

A cluster randomised trial (among 599 patients living with T2D attending clinics in the Tshwane District) found that screening for eye and feet complications was only reported in 8.2% and 6.5% of the patients, respectively.^31^ Based on the available guidelines, the recommended interval between HBA1c tests is 6 months.^11^ In this study, only 61% of HCPs indicated that the tests were performed in their facilities as recommended. A recent study in Tshwane Metropolitan Municipality found that HBA1c was performed on 72% of patients; however, it is not clear if these were done according to the recommended guidelines.^23^ Previous studies in South Africa, on the other hand, found that HBA1c was conducted on 23% and 29% of patients, respectively.^13,14^ And lastly, a study in Kinshasa indicated that 14.3% of clinics reported to have performed HBA1c tests on patients, in accordance with the prevailing guidelines.^25^ Overall, the results of this study demonstrated poor compliance to guidelines among HCPs regarding screening of chronic complications and HBA1c testing, which is in line with findings from the aforementioned studies.

### Individual and group education of patients living with type 2 diabetes mellitus in Tshwane

Patient education is widely recognised as the cornerstone of effective management of T2D among patients. By equipping patients with the necessary skills and knowledge, they are empowered to actively participate in their own care and adhere to healthy lifestyles. This will potentially reduce the risks of complications and ultimately improve their quality of life.^32,33,34,35^ Research from South Africa and Zimbabwe paints a concerning picture, revealing a widespread lack of adequate knowledge about T2D among patients.^36,37,38,39^ While this study did not directly assess the proportion of patients receiving diabetes education, a study conducted in three South African provinces found that only 22% of patients with T2D had access to health education.^29^ This highlights a significant gap in the current efforts and underscores the urgent need to prioritise patient education as a key strategy for improving T2D outcomes.^40^

In this study, 66% of HCPs indicated that they always provided individual education sessions to patients, while only 17% reported that group sessions were held with patients. A study in Tshwane showed that individual education of patients living with T2D was done in all clinics and 67% of the clinics reported to conduct group sessions with their patients.^17^ It is important to note that in a study conducted in Limpopo Province of South Africa, clinics organised support groups for chronic patients to reinforce compliance and management of their conditions; however, such sessions were not held in every clinic because most patients were not willing to be part of the groups.^30^

Regarding the HCPs who usually conduct patient education, the findings of this study revealed that nurses were mostly providing individual education. Contrary to our study, a study in Tshwane District found that education of patients living with T2D was offered mainly by health promoters.^17^ Interestingly, other studies have shown that diabetes education programmes provided by a PHC nurse or onsite teams of nurses and dietitians can be more effective in the control and management of T2D.^35,41^ Although nurses were found to be good health educators, it would be useful to understand the reasons for the supposed change of educators in the Tshwane district as was seen from this study and that of Webb and colleagues, which were conducted in the same district.^17^ Apart from poor adherence to guidelines for the care of patients in this study, is also the ability of HCPs to provide quality education considering their competing work-related priorities and patient workload. More studies are required to explore this matter in depth.

### Strengths and limitations

This study acknowledges the inherent limitations associated with self-reported data from HCPs, as this may not indicate the actual presence of equipment and other resources. While anonymity and confidentiality were ensured, social desirability and nonresponse bias could still influence responses. Additionally, the information provided was not validated through external means like documentation, observation or patient interviews, potentially affecting the accuracy of the findings.

### Recommendations

This study recommends that healthcare facilities be provided with adequate essential equipment, guidelines, regular in-service training and technology to assist HCPs to effectively manage and monitor patients living with T2D. One such technology is the use of real-time continuous glucose monitoring among patients with T2D, even if it is used intermittently. Several studies have shown the benefits of real-time continuous glucose monitoring which can assist in monitoring glycaemic control among patients, as poor glycaemic control has been linked to the development of complications.^42,43^ Concerning patient education, more studies are required to assess the quality of education provided to patients and the level of confidence among patients on self-management. Another form of technology that can be considered at the PHC level is the use of conversational agents as alternatives to traditional face-to-face provision of health education, which can alleviate the pressure on HCPs to offer education amid other treatment and care priorities.

## Conclusion

This study aimed to determine the care and management of patients living with T2D focusing on the availability of equipment, diabetes management guidelines, frequency of screening tests performed and provision of educational sessions to patients. The results – as reported by HCPs – revealed poor availability of equipment and treatment guidelines in PHC facilities (i.e. clinics and CHCs) in Tshwane. Poor availability of these equipment and guidelines corresponds with inadequate screening for chronic complications. Community health centres seem to be more affected than the clinics for reasons that are unclear at this point. Education of patients living with T2D seems inadequate with limited or no group sessions in most facilities. The lack of screening for chronic complications and inadequate patient education may result in missed opportunities for early diagnosis of complications which may require patients to be referred to higher levels of care. It is therefore important that secondary prevention efforts be prioritised to prevent the development of diabetes-related complications and improve the quality of life of patients.
